# Dutch randomized trial comparing standard catheter-directed thrombolysis versus Ultrasound-accElerated Thrombolysis for thromboembolic infrainguinal disease (DUET): design and rationale

**DOI:** 10.1186/1745-6215-12-20

**Published:** 2011-01-23

**Authors:** A Marjolein Schrijver, Michel MPJ Reijnen, Jacques A van Oostayen, Rudolf PJ Tutein Nolthenius, Pieter HM van der Valk, Arjan WJ Hoksbergen, Rutger J Lely, Bram Fioole, Dammis Vroegindeweij, Marc van Leersum, Jean-Paul PM de Vries

**Affiliations:** 1 Department of Surgery, St. Antonius Hospital Nieuwegein, The Netherlands; 2 Department of Surgery, Rijnstate Hospital Arnhem, The Netherlands; 3 Department of Radiology, Rijnstate Hospital Arnhem, The Netherlands; 4 Department of Surgery, Albert Schweitzer Hospital Dordrecht, The Netherlands; 5 Department of Radiology, Albert Schweitzer Hospital Dordrecht, The Netherlands; 6 Department of Surgery, Free University Medical Centre Amsterdam, The Netherlands; 7 Department of Radiology, Free University Medical Centre Amsterdam, The Netherlands; 8 Department of Surgery, Maasstad Hospital Rotterdam, The Netherlands; 9 Department of Radiology, Maasstad Hospital Rotterdam, The Netherlands; 10 Department of Radiology, St. Antonius Hospital Nieuwegein, The Netherlands

## Abstract

**Background:**

The use of thrombolytic therapy in the treatment of thrombosed infrainguinal native arteries and bypass grafts has increased over the years. Main limitation of this treatment modality, however, is the occurrence of bleeding complications. Low intensity ultrasound (US) has been shown to accelerate enzymatic thrombolysis, thereby reducing therapy time. So far, no randomized trials have investigated the application of US-accelerated thrombolysis in the treatment of thrombosed infra-inguinal native arteries or bypass grafts. The DUET study (Dutch randomized trial comparing standard catheter-directed thrombolysis versus Ultrasound-accElerated Thrombolysis for thrombo-embolic infrainguinal disease) is designed to assess whether US-accelerated thrombolysis will reduce therapy time significantly compared with standard catheter-directed thrombolysis.

**Methods/design:**

Sixty adult patients with recently (between 1 and 7 weeks) thrombosed infrainguinal native arteries or bypass grafts with acute limb ischemia class I or IIa, according to the Rutherford classification for acute ischemia, will be randomly allocated to either standard thrombolysis (group A) or US-accelerated thrombolysis (group B). Patients will be recruited from 5 teaching hospitals in the Netherlands during a 2-year period. The primary endpoint is the duration of catheter-directed thrombolysis needed for uninterrupted flow in the thrombosed infrainguinal native artery or bypass graft, with outflow through at least 1 crural artery.

**Discussion:**

The DUET study is a randomized controlled trial that will provide evidence of whether US-accelerated thrombolysis will significantly reduce therapy time in patients with recently thrombosed infrainguinal native arteries or bypass grafts, without an increase in complications.

**Trial registration:**

Current Controlled Trials ISRCTN72676102

## Background

Thrombosis of an infrainguinal bypass graft or the native lower leg arteries has been associated with a high rate of limb loss and significant morbidity and mortality [1]. Traditional therapy with thrombectomy and eventually additional (bypass) surgery has been associated with moderate long-term patency rates [2]. Catheter-directed thrombolysis was introduced in the early 1980s as a treatment option for occluded bypass grafts. This technique has advantages that make it ideal for acute occlusions, including avoidance of mechanical injury to the endothelium, no need for surgical interventions and more complete lysis of the clot in the bypass graft or outflow arteries. Moreover, it can dissolve platelet-fibrin aggregates in the microcirculation and thrombi in collateral vessels [3].

A recent systematic review that included a meta-analysis of 5 large randomized controlled trials with a total of 1283 patients comparing surgery with thrombolysis in the management of acute lower limb ischemia showed no significant difference between them in limb salvage or death at 30 days, 6 months, or 1 year. However, hemorrhagic complications and distal embolization were more likely at 30 days in thrombolysis patients (8.8% and 12.4%, respectively) than in surgery patients [4,5].

The incidence of these complications might be reduced by a reduction in thrombolytic therapy time. In recent years, ultrasound (US) has been used to accelerate thrombolysis. Low-intensity US-accelerated thrombolysis has been shown to accelerate enzymatic clot lysis in vitro by loosening fibrin strands and thereby increasing thrombus permeability and exposing more plasminogen receptors for binding, without mechanically disrupting the clot [6-8]. The safety of this technique has been shown in the treatment of embolic stroke [9] and deep venous thrombosis [10]. Only two prospective series have been published on the use of US-accelerated thrombolysis in the treatment of thromboembolic obstructions of native arteries and bypass grafts of the lower extremities. In a recent study by Wissgott et al, 25 patients with acute obstructions of the native lower limb arteries were treated with US-accelerated thrombolysis. The technical success rate of 100%, and total clot removal was achieved in 88% of the patients within 24 hours. There were no complications related to the catheter system. At the 1-month follow-up, 2 reocclusions had occurred [11]. Motarjeme et al used US-accelerated thrombolysis in the treatment 24 arterial occlusions and 12 venous occlusions, and complete lysis was achieved in 23 (96%) arterial occlusions. Average time to achieve complete lysis was 16.4 hours (range, 3-25 hours). During the 12-month follow-up, only 1 femoropopliteal graft rethrombosed. No bleeding or other complications occurred [12].

So far, no randomized trials have focused on US-accelerated and standard catheter-derived thrombolysis for arterial thromboembolic disorders of the lower limbs. The DUET (Dutch randomised trial comparing standard catheter-directed thrombolysis versus Ultrasound-accElerated Thrombolysis for thromboembolic infrainguinal disease) study is designed to compare US-accelerated thrombolysis with standard thrombolysis in patients with recently thrombosed infrainguinal native arteries or bypass grafts.

### Hypothesis

We anticipate that US-accelerated thrombolysis will result in a significant reduction in thrombolysis time (12 hours or more), without increasing the complication rate.

## Methods/Design

### Study objectives

The purpose of the study is to demonstrate that US-accelerated thrombolysis will significantly reduce (by at least 12 hours) therapy time compared with standard thrombolysis, without increasing complication rate.

### Primary endpoint

The primary end point is the duration of catheter-directed thrombolysis needed for uninterrupted flow in the thrombosed infrainguinal native artery or bypass graft with outflow through at least 1 crural artery.

### Secondary endpoints

1. Technical success, defined as complete lysis of the thrombus of the native artery or bypass graft, without distal thromboembolic complications

2. Number of units of urokinase needed for uninterrupted flow in the thrombosed infrainguinal native artery or bypass graft with outflow through at least 1 crural artery

3. Thrombolysis-induced hemorrhagic complications

4. Thirty-day mortality

5. Duration of hospital admission

6. Costs of hospital admission

7. Thirty-day patency of the target native artery or bypass graft, as evidenced by magnetic resonance angiography (MRA)

8. Drop of serum fibrinogen concentration to below 1.0 g/L during procedure

9. Conversion to open surgery

10. Distal thromboembolic complications

11. Other complications

### Definitions

Complete lysis: clot lysis of >95% determined by arteriographic measurements of the thrombosed native arterial segment or bypass graft [13].

Hemorrhagic complications are defined as:

• hemorrhagic complication requiring interruption or ending of thrombolysis

• hemorrhagic complication requiring surgical intervention

• hemorrhagic complication requiring transfusion

Rutherford classification of acute limb ischemia [14]:

Class I: Viable--not immediately threatened, no sensory loss or muscle weakness, arterial Doppler signal is audible

Class IIa: Marginally threatened--salvageable if promptly treated, minimal sensory loss, no muscle weakness, arterial Doppler signal is often inaudible

Class IIb: Immediately threatened--salvageable with immediate revascularization, sensory loss associated with rest pain in more than the toes, mild to moderate muscle weakness, arterial Doppler signal is usually inaudible.

Class III: Irreversible--major tissue loss or permanent nerve damage inevitable if there is significant delay before intervention, profound limb anaesthesia and paralysis, arterial and venous Doppler signal is inaudible

### Design of study

This is a multicenter 2-group parallel randomized trial.

### Participating centres

Participating centers are Albert Schweitzer Hospital Dordrecht, Free University Medical Centre Amsterdam, Maasstad Hospital Rotterdam, Rijnstate Hospital Arnhem, and St. Antonius Hospital Nieuwegein.

### Setting

Patients who will meet the inclusion criteria will be enrolled from 5 teaching hospitals in the Netherlands.

### Patients

A total of 60 adult patients with acute lower limb ischemia due to recently (between 1 and 7 weeks) thrombosed infrainguinal native arteries or bypass grafts will be randomized.

### Eligibility criteria

#### Inclusion criteria

1. Men and women older than 18 years and younger than 85 years old

2. Patients with recently (between 1 and 7 weeks) thrombosed femoropopliteal or femorocrural native arteries or femoropopliteal or femorocrural venous or prosthetic bypass grafts with ischemic complaints

3. Patients with acute lower limb ischaemia class I and IIa according to the Rutherford classification

4. Patients understand the nature of the procedure and provide written informed consent before enrollment in the study

#### Exclusion criteria

1. Patients with isolated common femoral artery thrombosis, including the origin of the superficial femoral artery and profunda femoral artery

2. Patients with localized (less than 5 cm) emboli or occlusions in the native femoropopliteal arteries

3. Patients with clinical complaints of acute lower limb ischemia due to thrombosis of the femoropopliteal or femorocrural native arteries, or femoropopliteal or femorocrural venous or prosthetic bypass grafts less than 1 week and more than 7 weeks

4. Patients with acute lower limb ischemia class IIb and III according to the Rutherford classification

5. Patients for whom antiplatelet therapy, anticoagulants, or thrombolytic drugs are contraindicated

6. Recent (less than 6 weeks) ischemic stroke or cerebral bleeding

7. Patients with recent (less than 6 weeks) surgery

8. Severe hypertension (diastolic blood pressure greater than 110 mm Hg, systolic blood pressure greater than 200 mm Hg)

9. Current malignancy

10. Patients with a history of prior life-threatening reaction to contrast medium

11. Patients with uncorrected bleeding disorders (gastrointestinal ulcer, menorrhagia, liver failure)

12. Women with child-bearing potential not taking adequate contraceptives or currently breastfeeding

13. Pregnancy

14. Patients considered hemodynamically unstable at the onset of the procedure

15. Patients who refuse treatment

16. Patients who are currently participating in another investigational drug or device study who have not completed the entire follow-up period

17. Patients younger than 18 years or older than 85 years

18. Severe comorbid condition with a life expectancy of less than 1 month

19. Contraindication for magnetic resonance imaging (MRI)

### Randomization

Central randomization will take place using a computerized randomization procedure. Block-randomization is used and stratified according to whether a native artery or a bypass graft is involved. Blinding will not be used.

### Ethics

This study is conducted in accordance with the principles of the Declaration of Helsinki and Good Clinical Practice guidelines. The study protocol was approved by the Ethics Committee (METC) of the St. Antonius Hospital Nieuwegein (R-09.17A). Written informed consent will be obtained from all patients, before randomization.

### Safety and quality control

#### Data Safety Monitoring Board

The Data Safety Monitoring Board (DSMB) is composed of 4 members: 2 independent vascular surgeons and 2 independent interventional radiologists. None of the 4 members is working in one of the hospitals that will include patients. The role of the DSMB is to review safety and to make recommendations regarding the conduct of the study to the steering committee and to the accredited METC that approved the study protocol.

#### Adverse and serious adverse events

Adverse events (AE) are defined as any undesirable experience occurring to a participant during the study, whether or not considered related to the investigational device. This definition includes events occurring during hospital stay right up to 30 ± 7 days of follow-up. Underlying disease that was present at the time of enrollment is not reported as an AE, but any increase in the severity of the underlying disease will be reported as an AE.

All AEs will be monitored from the time of enrolment through the 30-day follow-up visit. AEs can be classified as moderate or serious and will be recorded on the case record forms (CRFs). A description of the event, including the start date, end date, whether device-related, any action taken, and the outcome will be provided along with the investigator's assessment of the relationship between the AE and the study treatment.

A serious adverse event (SAE) is any untoward medical occurrence or effect that at any dose results in death, or that;

• is life-threatening at the time of the event;

• requires hospitalization or prolongation of an existing inpatient's hospitalization;

• results in persistent or significant disability or incapacity; or

• necessitates an intervention to prevent a permanent impairment of a body function or permanent damage to a body structure.

Clinical events to be considered and reported as SAEs include:

• death

• myocardial infarction

• stroke

• bleeding complication requiring interruption or ending of thrombolysis

• bleeding complication requiring surgical intervention

• bleeding complication requiring transfusion

A moderate adverse event (MAE) is any untoward medical occurrence or effect that at any dose will not lead to death, or

• life-threats,

• significant disability or incapacity,

• require hospitalization or prolongation of an existing inpatient's hospitalization, or

• necessitate an intervention to prevent a permanent impairment of a body function or permanent damage to a body structure.

Clinical events to be considered and reported as MAEs include:

• groin hematoma,

• bleeding complication not requiring interruption or ending of thrombolysis, surgical intervention, or

• transfusion.

Data on SAEs and MAEs will be reported to the DSMB and to the accredited METC via the "Toetsingonline" website of the website of the Central Committee on Research inv. Human Subjects (CCMO, ccmo.nl).

### Statistical analysis

#### Intention-to-treat

The analysis will be performed in accordance with intention-to-treat principle.

#### Sample size calculation

The assumption has been made that the mean duration for successful standard thrombolytic treatment of infrainguinal native arteries or bypass grafts is 2.5 ± 1 day, based on retrospective series from the St. Antonius Hospital, Nieuwegein. Given a power of 90% and a 2-tailed significance of 5%, a 2-armed randomized trial including 26 patients in each arm will be needed to prove a significant reduction in therapy time (12 hours or more) with US-accelerated thrombolysis compared with standard thrombolysis. Taking into account 5% to 10% dropouts in each arm, 30 patients need to be included in each study arm. We will analyze the primary outcome by means of Kaplan-Meier and Log-rank test. The sample size calculation is also based on this premise. We will assess imbalance in prognostic factors as a secondary analysis by means of Cox-proportional Hazards multivariate analysis.

An interim analysis on the primary end point (ie, efficacy) will be performed after 50% of patients have completed their follow-up. The Peto approach will be followed, meaning that the study will only be stopped for beneficial effects in case of a *P *< .001 [15]. The study will not be stopped in case of futility. End points in blinded groups will be assessed by the DSMB.

### Intervention

#### Group A (standard thrombolysis)

Standard thrombolysis will be performed with use of a 5F UniFuse Infusion catheter (AngioDynamics, Queensbury, NY, USA) with a working length of 90 cm, with infusion holes around the circumference of the distal 10 cm to 50 cm of the catheter. Access sites may be the contralateral common femoral artery, the ipsilateral common femoral artery, or a brachial approach.

During the initial angiography, the catheter will be navigated over a guidewire and positioned in such way that the entire distal segment with the infusion holes is in the thrombosed segment. At standardized intervals, a control angiography will be performed. During each control angiography, the distal part of the thrombolysis catheter will be repositioned in the remaining thrombosed segment.

#### Group B (US-accelerated thrombolysis)

US-accelerated thrombolysis involves simultaneous delivery of low-intensity US and a thrombolytic agent into a thrombosed vessel. US-accelerated thrombolysis will be performed with use of the EKOS EndoWave system (EKOS Corporation, Bothell, WA, USA). This system consists of a 5.2F multilumen thrombolysis delivery catheter with a 106 cm or 135 cm working length and a matching US coaxial core wire with a working zone of 6 cm to 50 cm. The central lumen of the multilumen thrombolysis delivery catheter accommodates the US core. Thrombolytics will be infused through 3 drug lumens containing multiple side holes. Low-intensity (2.2 MHz), high-frequency US will be delivered over the entire length of the infusion catheter. US power and local temperature are automatically controlled by a portable control unit.

During the initial angiography, a multilumen thrombolysis delivery catheter will be navigated over a guidewire into the thrombosed segment in such a way that the US treatment zone traverses the entire thrombosed segment and the tip of the infusion catheter is located distal to the thrombosed segment. After final positioning, the guidewire will be exchanged for a matching US core wire, and thrombolytic therapy will be started. Likewise, a control angiography will be performed at standardized intervals.

If placement of the EKOS system is technically not feasible, the procedure will be defined as a technical treatment failure. The vascular surgeon or interventional radiologist, or both, may decide what alternative treatment they will start, for instance, standard thrombolysis or conversion to open surgery. The outcome of these patients will be analyzed in group B according to the intention-to-treat principles.

#### General

The primary angiography in groups A and B will be started at day 1 at 8.00 a.m. ± 1 hour for logistic reasons. After successful positioning of the thrombolysis catheter, a bolus of 250,000 IU urokinase is given and followed by a continuous infusion with a dose of 100,000 IU urokinase/h. A control angiography will be performed every 6 ± 1 hours. During the night, standard angiography will only be performed in emergencies. Angiographies will be performed the next day at 8.00 a.m. ± 1 hour; 2.00 p.m. ± 1 hour, and 8.00 p.m. ± 1 hour. A checklist is completed during each angiography.

The pressure curve from the EKOS monitor will be recorded constantly from the start of the procedure. From the start of thrombolysis until completion angiography, the ankle-brachial indices of the treated leg and a standardized pain score will be recorded every 3 hours by a nurse practitioner or surgical resident. Every day during thrombolysis, the fibrinogen concentration must be checked: if <1.0 g/L the urokinase rate must be lowered to 50,000 IU/h; if the fibrinogen concentration is <0.5 g/L, thrombolysis must be stopped. If the obstructive clot has been successfully thrombolized, any necessary additional procedure, for instance percutaneous transluminal angioplasty (PTA) of the inflow or outflow artery, is performed before the completion angiography, and again, a checklist of the last angiography is completed.

After successful thrombolysis, systemic heparin is given (25,000 IU/24 hours) and coumarin derivatives are started. Activated partial thromboplastin time (aPTT) will be measured daily during heparinization. The target international normalized ratio (INR) will be 2.5 to 3.5; if this value is reached, heparinization can be stopped.

### Data collection

Data will be collected by means of a CRF during treatment in the participating centers. The CRF will be completed prospectively during hospital admission and during follow-up. After that, the CRFs will be forwarded to the data coordinating center. There will be regular contact between the study coordinators and the participating centers.

### Follow-up

Patients are followed-up during their hospital stay. At 30 ± 7 days after thrombolysis has been completed, there is one follow-up visit in the outpatient department, including a physical examination, ankle-brachial index, treadmill test, and contrast-enhanced MRA of the treated leg, according to current guidelines [16]. During follow-up, any additional procedures, conversion to open surgery, duration and costs of hospital admission, death, distal thromboembolic complications, and other complications will be recorded.

## Discussion

Several treatment strategies have focused on accelerating thrombolytic therapy to improve its effectiveness and to reduce treatment time and, therefore, adverse effects. US-accelerated thrombolysis, which involves the simultaneous delivery of low-intensity US and a thrombolytic agent into a thrombosed vessel, has been shown to reduce therapy time. The DUET study is designed to compare US-accelerated thrombolysis with standard thrombolysis in patients with recently thrombosed infrainguinal native arteries or bypass grafts.

Only patients with acute lower limb ischemia in class I and IIa, according to the Rutherford classification, will be included in our study, because catheter-directed thrombolysis is the initial treatment chosen in patients with viable or minimally threatened lower limbs. Immediate surgical revascularization is indicated in most patients with acute lower limb ischemia class IIb and III; therefore, these patients will be excluded from the study.

Prolonged thrombolysis therapy might be expected in clots older than 1 week. Most clots that are less than 1 week old can be rather easily and quickly treated with standard catheter-directed thrombolysis or thrombosuction. The additional value of the more expensive US-accelerated thrombolysis in these thrombi may be argued and these are therefore excluded from this study.

Randomization is stratified according to whether a native artery or a bypass graft is occluded, because it has been shown that thrombolysis of a bypass graft will lead to better outcome than thrombolysis of a native artery.

To test our hypothesis, a control angiography should be performed every 12 hours. Therefore, our sample size calculation is based on a reduction in therapy time of 12 hours or more. Control angiographies at 8:00 a.m. and at 8.00 p.m. would have been sufficient. However, in our study protocol we have incorporated an extra control angiography at 2.00 p.m. to measure our primary outcome more precisely and to reduce any unnecessary exposure to thrombolytics in our study participants.

Two vascular surgeons and 2 interventional radiologists from 2 nonparticipating centers will review all angiographies independently. The vascular surgeons and the interventional radiologists will be blinded for patient characteristics and for the results of the initial reports. They will be asked to classify the degree of lysis, ranging from no lysis, partial lysis, to complete lysis (>95% lysis).

If thrombolysis of the occluded segment is successful, any hemodynamically significant underlying atherosclerotic lesion will be identified and treated. Preferably, the underlying lesion will be treated endovascularly, with PTA with or without stent placement, or both. It will be the vascular surgeon's or interventional radiologists' decision to convert to open surgery if endovascular treatment of the underlying lesion is not feasible or if thrombolysis of the occluded segment is not successful.

## Conclusion

The DUET study is a randomized controlled trial that will provide evidence whether US-accelerated thrombolysis will significantly reduce therapy time compared with standard thrombolysis in patients with recently thrombosed infrainguinal native arteries or bypass grafts, without an increase in the number of complications.

## Competing interests

The authors declare that they have no competing interests.

## Authors' contributions

AMS and JPPMdV drafted the manuscript.

AMS, MMPJR, RPJTN, AWJH, MvL and JPPMdV participated in the design of the study.

All authors edited the manuscript and read and approved the final manuscript.

## Appendix

### DUET committee members

#### Steering committee

JPPM de Vries (chairman), AM Schrijver (principal investigator), M van Leersum, St. Antonius Hospital, Nieuwegein; MMPJ Reijnen, Rijnstate Hospital, Arnhem.

#### Monitoring Committee

GJ de Borst (chairman), EJ Vonken, UMC Utrecht; BP Keller, H Alkefaji, Martini Hopsital, Groningen.
